# A Review on T Cell Epitopes Identified Using Prediction and Cell-Mediated Immune Models for *Mycobacterium tuberculosis* and *Bordetella pertussis*

**DOI:** 10.3389/fimmu.2018.02778

**Published:** 2018-11-29

**Authors:** Yuan Tian, Ricardo da Silva Antunes, John Sidney, Cecilia S. Lindestam Arlehamn, Alba Grifoni, Sandeep Kumar Dhanda, Sinu Paul, Bjoern Peters, Daniela Weiskopf, Alessandro Sette

**Affiliations:** ^1^Division of Vaccine Discovery, La Jolla Institute for Immunology, La Jolla, CA, United States; ^2^Department of Medicine, University of California San Diego, La Jolla, CA, United States

**Keywords:** epitope, T cell, HLA, bacteria, *Mycobacterium tuberculosis*, *Bordetella pertussis*

## Abstract

In the present review, we summarize work from our as well as other groups related to the characterization of bacterial T cell epitopes, with a specific focus on two important pathogens, namely, *Mycobacterium tuberculosis* (*Mtb*), the bacterium that causes tuberculosis (TB), and *Bordetella pertussis* (BP), the bacterium that causes whooping cough. Both bacteria and their associated diseases are of large societal significance. Although vaccines exist for both pathogens, their efficacy is incomplete. It is widely thought that defects and/or alteration in T cell compartments are associated with limited vaccine effectiveness. As discussed below, a full genome-wide map was performed in the case of *Mtb*. For BP, our focus has thus far been on the antigens contained in the acellular vaccine; a full genome-wide screen is in the planning stage. Nevertheless, the sum-total of the results in the two different bacterial systems allows us to exemplify approaches and techniques that we believe are generally applicable to the mapping and characterization of human immune responses to bacterial pathogens. Finally, we add, as a disclaimer, that this review by design is focused on the work produced by our laboratory as an illustration of approaches to the study of T cell responses to *Mtb* and BP, and is not meant to be comprehensive, nor to detract from the excellent work performed by many other groups.

## Epitope identification for mycobacteria

Our group has a long-standing interest in tuberculosis (TB). Early studies characterized a DR17-restricted epitope (1) and the effect of different T cell subsets on intracellular infection (2, 3). More recent studies characterized CD8 T cells (4). Subsequent studies described the first, to the best of our knowledge, true genome-wide screen for CD4 T cell epitopes (5). Subsequent studies report that CD4 T cells can recognize and target epitopes derived from mycobacterial ribosomal proteins and provide protective functions (6, 7).

The epitopes were further characterized by studies on the recognition of *Mycobacterium tuberculosis* (*Mtb*)-derived epitopes in a cohort with latent *Mtb* infection (8) and in diverse populations from five continents (9) and intragenus conservation (10). We presented an analysis of the complexity of *Mtb*-specific epitopes in *Mtb* infected South Africans (11) and provided evidence that bi-allelic RORC mutations are detrimental to host immunity against *Mtb* (12). We further showed that transcriptomic analysis revealed novel immune signatures associated with TB (13–15) and the differentiation and function of T cells are influenced by the availability antigens (16).

In particular, previous studies (5) demonstrated the feasibility of utilizing genome-wide screen to identify human leukocyte antigen (HLA) class II epitopes derived from *Mtb*, based on combined bioinformatic predictions and high throughput *ex vivo* ELISPOT assays. Feasibility of the approach had previously been demonstrated for viral targets, however tackling a bacterial genome expressing over 4,000 open reading frames (ORFs) had not been attempted. Genome-wide screens have also been conducted to identify CD8 T cell Mtb epitopes (17–20). Notably, immunodominant CD8 T cell epitopes are enriched in cell wall and secreted proteins (18, 19). Future studies will utilize the same approach to focus on *Bordetella pertussis* (BP), which causes whooping cough.

## Epitope identification for other bacteria especially BP

While our initial focus was mostly directed toward the study of *Mtb*, other microbes have also been studied. Maybeno et al. (21) described *Salmonella* epitopes, and Cannella et al. reported studies in *Brucella* (22). In the context of BP, we showed that initial whole-cell pertussis (wP) vaccination results in long-term Th1/Th17 polarization even with subsequent acellular boosters (23, 24).

It is hypothesized that the recent reemergence of BP infection is linked to the adoption of acellular pertussis (aP) vaccines based on specific BP antigens (FHA, Fim2/3, PRN, and PT). It is possible that the previous whole cell inactivated (wP) vaccine elicited a broader reactivity and targeted additional antigens, some of which might be of particular relevance and linked to superior vaccine performance. The extent and targets of T cell immunity in the context of natural infection and clinical disease are likewise not yet defined in a comprehensive fashion.

These considerations argue for performing broad epitope identification and characterization studies in BP as well. In the following sections we describe the techniques we have developed for the purpose of epitope identification and characterization, and then we describe specific applications to the TB and BP systems.

## Measuring HLA epitope affinity

Activation of alpha/beta classical T cells in general requires recognition of a specific peptide epitope, bound to specific major histocompatibility complex (MHC) molecules, a phenomenon classically named “HLA-restriction.” The methods used to establish restriction are described in a separate section below. Here we focus on the fact that, since HLA binding is a prerequisite for a peptide being actually recognized as an epitope, measuring its HLA binding affinity is a powerful method to select epitope candidates. The relevant quantitative binding thresholds have been defined for both class I (25) and class II (26–28).

Our group has been a pioneer in the development of techniques to measure the binding of peptides to MHC molecules, termed HLA molecules in humans. Over the course of the last 30 years we have measured almost half a million MHC peptide binding constants for over 100,000 peptide/MHC combinations, and our group contributed a chapter describing our assay platform in detail to the laboratory compendium *Current Protocols in Immunology* (29).

**Figure 1:**
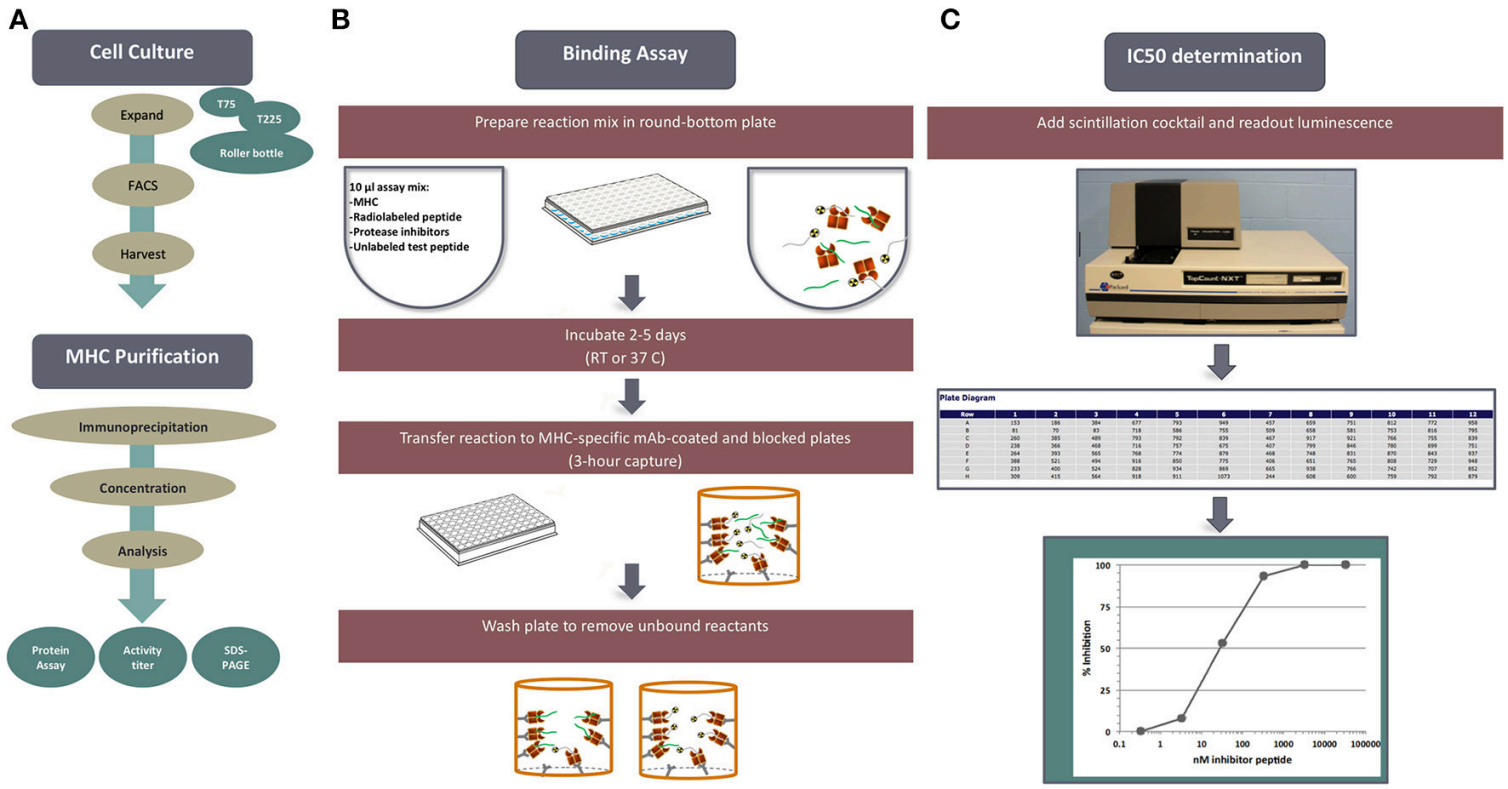
MHC-peptide binding assays. MHC binding affinities are determined in classical competition assays utilizing purified MHC molecules and high affinity radiolabeled peptide probes. **(A)** Is an overview, outline tissue culture, MHC purification, binding assay and readout. **(B)** Diagrams the set-up and performance of a competition assay. **(C)** Depicts read-out of signal using the TopCount plate reader and determination of peptide binding affinity.

The results obtained with this assay have been published in several 100 different peer reviewed journal articles. Our current assay panel allows measurements of binding to over 40 different HLA class I molecules and 35 HLA class II molecules. MHC binding is evaluated using a classical competition assay where peptides of interest competes with radiolabeled probe peptide for MHC binding (Figure 1). Plenty of supply of purified MHC molecules as well as labeled and unlabeled peptides is necessary for the establishment and usage of an MHC-peptide binding assay. Thus, our immunochemistry group has established an ongoing operation where cell lines expressing different alleles are expanded to allow for large-scale HLA purification by affinity chromatography.

Each cell line is rigorously characterized by HLA typing to ensure identity of the HLA allelic variant, and expression is monitored by flow cytometry. Standardized MHC purification protocols using affinity chromatography are in place. Furthermore, the purity and quantity of purified products are assessed by gel filtration and bicinchoninic acid assay (BCA) assays on each preparation. High affinity probes specific for each HLA have been identified for each allelic variant and used in a classic quantitative receptor ligand inhibition assay where IC50 values are used to approximate true K_D_ values. Bound and unbound radiolabeled peptides are separated following incubation for 2 days, and their relative abundance is quantified. The specificity of each of the assays was rigorously determined by demonstrating high affinity binding of known independently defined eluted peptides or T cell epitopes restricted by the allelic variant in question.

## HLA polymorphism and binding promiscuity

Thousands of different class I and class II HLA types exist in human populations. Most of the polymorphic residues line specific pockets in the HLA, which are involved in the peptide-HLA binding interaction. Accordingly, different HLA molecules have, in general, different binding specificities or “motifs” (30, 31). In fact, the capacity of general populations to bind a plethora of different sequences is the evolutionary force driving HLA polymorphism, to oppose the potential of pathogens to escape immune recognition by avoiding presentation of their peptides.

This extensive polymorphism poses a fundamental challenge for epitope identification and validation. A comprehensive effort targeting thousands of variants would be impractical and unfeasible. A simple pragmatic solution would be to target HLA alleles that are present with the highest frequencies. However, this approach has to factor the reality that the frequencies of different HLA can vary dramatically across different ethnicities. Thus, comprehensive coverage of ethnically diverse populations requires careful analysis.

A phenomenon that counterbalances these difficulties and was originally recognized as a means to simplify the task is linked to HLA promiscuity. Indeed, it was already observed by the early 1990s that the same peptide could bind and be recognized by multiple HLA types (32–37), denoted as epitope “promiscuity.” Subsequent studies demonstrated that both classes I and II HLAs can be grouped into supertypes, defined on the basis of overlapping peptide binding repertoires (38–46). This is specifically applicable to HLA class II of the DR loci (33, 47, 48), and also DP (27, 49–51) and DQ (26, 51, 52).

A subsequent study used binding data of HLA DR, DQ, and DP to quantitatively assess how much promiscuity existed in HLA class II molecules. We found that these HLAs could be divided into seven major supertypes and, rather surprisingly, the repertoire overlap of class II supertypes was five to ten-fold higher than that of class I supertypes (51). These results indicated that if promiscuous binding would translate into promiscuous T cell recognition, then promiscuous epitopes might constitute a significant proportion of the total response.

## Promiscuous HLA class II epitopes

In several independent studies and antigen systems we tested the hypotheses that promiscuous epitopes account for a significant fraction of the specific immune response and that promiscuous epitopes can be identified by bioinformatic approaches. The notion that HLA class II promiscuous epitopes correspond to dominant epitopes accounting for a large fraction of the antigen specific response was initially evaluated with a set of overlapping 15-mer peptides spanning the Erythropoietin (EPO) protein (53). A large volume of subsequent data has demonstrated that these findings are generalizable to other systems, including proteins derived from infectious agents and allergens.

One series of experiments analyzed in detail the reactivity of allergic donors to the common allergen timothy grass (*Phleum pretense*), a mediator of hay fever (54). Over 40 different epitope regions were recognized, but upon closer inspection it was determined that only *nine* of them were required to cover 51% of the total response. These dominant regions were shown to correspond to promiscuously recognized epitopes, and shown to be predicted by bioinformatics algorithms targeting the most common DR, DP, and DQ variants.

This result was not limited to the timothy grass system. Indeed, similar results were obtained in several different allergen systems, including the *Blatella germanica* (Bla g) antigens associated with cockroach allergies (55). And, in a broader study, a panel of 133 allergens derived from 28 different sources, including fungi, trees, grasses, weeds, and indoor allergens, was surveyed utilizing predicted promiscuous HLA class II-binding peptides and ELISPOT assays with PBMC from allergic donors, resulting in the identification of 257 T cell epitopes (56).

In conclusion, a number of studies have shown that many peptides with highly promiscuous binding capacity are frequently recognized by immune individuals, and that promiscuous recognition in the context of multiple HLA class II molecules may be a mechanism significantly contributing to epitope immunodominance (33, 53, 57–60). This might be related to the fact that promiscuous epitopes tend to bind HLA with high affinity, or simply that binding to multiple HLAs gives an epitope multiple contexts where it can be associated with immunogenicity. Several studies by our group have demonstrated that bioinformatic predictions directed toward selection of the most promiscuous binding peptides can identify a significant fraction of the pathogen or allergen specific response (54–56, 61).

## Development of tools to predict promiscuous epitopes

**Figure 2:**
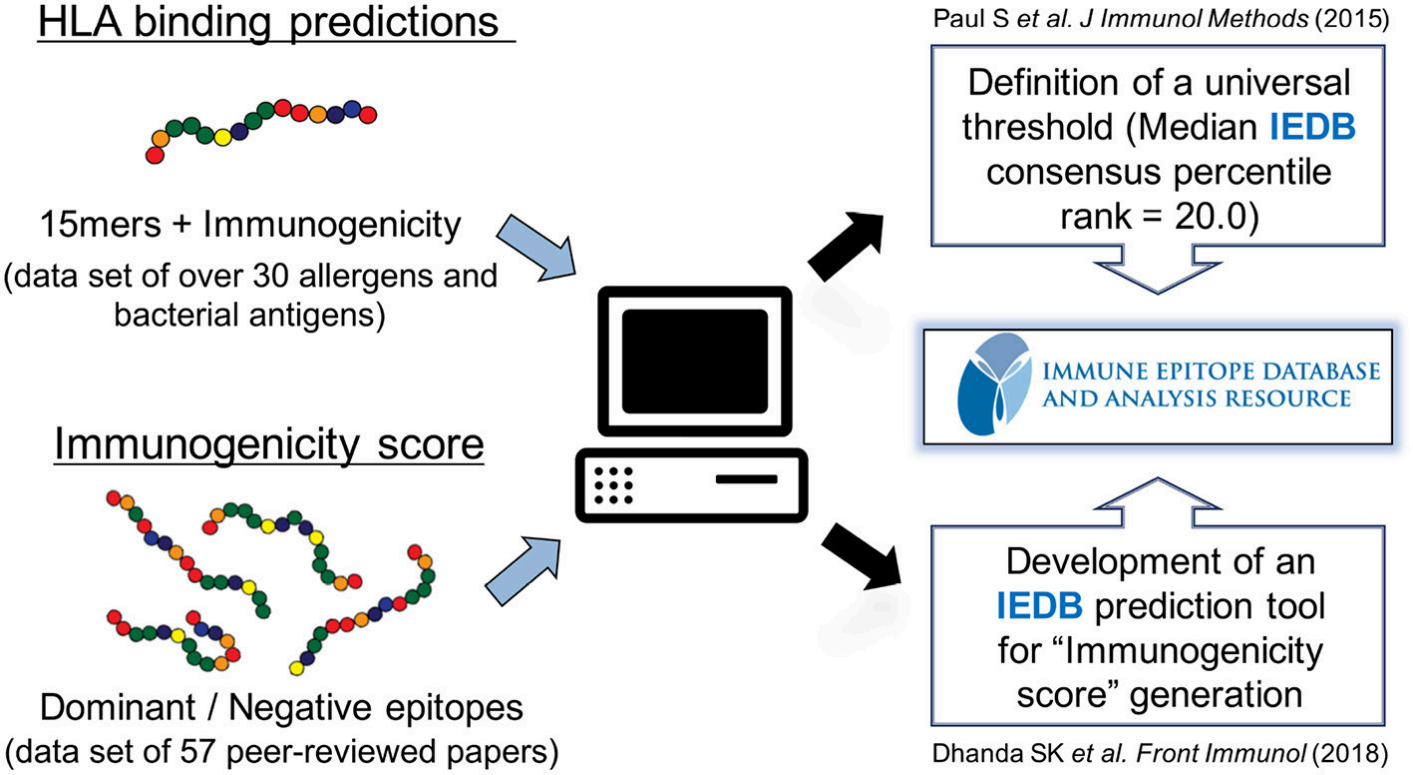
Prediction of HLA class II-restricted T cell epitopes. A strategy to globally predict epitopes recognized by human populations has been developed and validated using HLA class II binding prediction tools from the Immune Epitope Database and Analysis Resource (IEDB). A consensus percentile rank of ≤ 20 has been established. In addition, an artificial neural network model using sets of dominant epitopes and negative peptides has been built to generate “immunogenicity score” that predicts CD4 T cell immunogenicity in the absence of HLA data.

In the next series of investigations, we sought to derive and optimize a universal prediction schema based on data where sets of 15-mers overlapping by 10 amino acids representing the entire sequences of over 30 different allergens and bacterial proteins had been tested for T cell reactivity in human patients (62) (Figure 2). We specifically wanted to answer the question of how many predictions needed to be combined for maximal efficacy in real human patient populations, and furthermore, we wanted to determine which specific alleles should be included in such an optimal prediction tool. We defined optimal prediction parameters, and the resulting strategy was validated using a blind set of immunogenicity data that had not been utilized to derive the prediction scheme. We found that a 20th percentile IEDB consensus rank, combining predictions for a particular set of seven HLA class II, can predict about half of the total response. This approach can therefore be utilized as a universal prediction scheme, as it has been validated in a broad set of antigenic systems and in genetically diverse human patient populations.

In the study referenced above, the use of actual binding data instead of predictions did not improve the efficacy of the scheme, nor did performing allele specific predictions based on the particular HLA expressed in each individual patient. This indicated that, as expected the limited efficacy of the prediction was not due to the limitations of the algorithms, but rather that HLA binding predictions are associated with a rather high false positive rate, which fits well with the understanding that HLA binding is necessary but not sufficient for immunogenicity. Thus, other factors related to T cell repertoire and antigens processing also play a prominent role.

To address this issue, we used matched sets of dominant epitopes and negative peptides curated from the literature to train neural networks (63). The resulting “immunogenicity score” was further validated on 57 additional datasets (Figure 2). In all, data derived from more than 1,500 human donors and 2,000 peptides was considered in this training and validation effort. The results demonstrated that this agnostic “immunogenicity score” was effective in predicting dominant epitopes and human immunogenicity data. Surprisingly, the combination of immunogenicity score and HLA promiscuous predictions was associated with limited overall predictive improvement, suggesting, as previously noted, that antigen processing/T cell repertoire selection and HLA binding capacity might be influenced by coordinate evolution (64). Taken together, these results highlight that the bioinformatic tools necessary to identify promiscuous epitopes are available and have been validated in several independent studies, in different antigen system and different ethnicities.

## Determining HLA restriction of T cell responses

Determination of HLA restriction is a key element of epitope characterization, and precise knowledge of HLA restriction is also necessary to derive tetrameric staining reagents. HLA restriction was originally determined by the use of antibodies specific for different HLAs, in conjunction with antigen presentation assays; the essence of this strategy is to identify an antibody that blocks presentation of a given peptide to a given defined source of responding T cells (60). While straightforward in principle, this assay is often challenging, since antibodies with suitable specificity and selectivity are often not available, and T cells might promiscuously recognize the same peptide presented by multiple HLAs, yielding results difficult to interpret. Furthermore, the antibody and epitope concentration in the assay must be carefully controlled as excessive amounts of antibodies will inactivate the antigen-presenting cells (APCs) non-specifically, and high concentration of epitope will lead to self-presentation from the responding T cells.

An alternative is represented by determining whether the peptide can be presented by panels of partially HLA matched/mismatched cell lines/PBMCs. This is a powerful and simple approach, but can be limited by availability of suitable cell lines, and complicated again by promiscuous presentation, and the fact certain HLA combinations are in tight linkage disequilibrium. To overcome this limitation, we described an approach to define HLA class II restriction covering DP, DQ, and DR allelic variants that are most commonly represented in the general population (65). We specifically selected 46 DP, DQ, and DR HLAs which were projected to cover ~90% of these loci and constitute >66% of all the genes at each of these loci. Utilizing HLA data of actual populations from different geographical locations in the USA and Africa, we verified that these projections were accurate. A panel of single HLA transfected cell lines was developed and validated in a series of experiments, involving assessing HLA expression, identity, peptide binding, and epitope presentation (65).

The utility of this panel was further demonstrated by a quantitative study of HLA restriction and antigen-specific responses in a cohort of Mtb-immune individuals (11). Using APCs transfected with the panel of HLA class II molecules described above, HLA restrictions for nearly 300 different epitope/donor combinations were mapped. These results were the first large scale estimate of epitope complexity of CD4 T cell responses in a patient population and a microbial human pathogen, and indicated that the majority of epitopes were associated with promiscuous HLA restriction, further demonstrating the feasibility of the approach developed.

As an alternative complementary approach, we developed a method called Restrictor Analysis Tool for Epitopes (RATE) that can infer HLA restriction using CD4 T cell response data from HLA-typed individulas (66), The method, available online in the IEDB analysis resource, starts by inspecting, one epitope at a time, the HLA types present in individuals responding to each epitope. Then for each of these HLAs, calculates those enriched in frequency, comparing responders and non-responders to the specific epitope. The automated calculation yields a table of likely restrictions, Odd Ratios (ORs) and associated *p*-values. The method was validated by various experimental approaches, which derived strategies and thresholds for optimal performance (66, 67). The method is most effective for monogamous restrictions and by definition less able to detect promiscuous restrictions and HLA frequency variations due to genetic linkage.

## Analysis of epitope conservation

Several lines of evidence indicate that sequence variability and conservation can have a dramatic effect on the shaping and effectiveness of immune responses in general and T cell responses in particular. This influence is dynamic and can have both positive and negative effects.

One broad series of effects relates to immunological pressure exerted by antimicrobial responses, of which probably amongst the most well noted cases are the widespread mutation of T cell epitopes observed in HIV and HCV (68, 69). It has to be underlined that pathogen escape by mutation is most effective for microbes with small genomes, or with responses of limited breadth, since simultaneous escape of a responses directed against large genomes and a large number of antigens/epitopes is by definition unlikely. It has indeed been proposed that the switch from wP to aP generated a response that is less diverse and created an opportunity for BP to escape vaccine responses (70–72). It will be important to compare mutation rates of epitopes and non-epitopes, in wP, and aP antigens, to potentially either refute or support this hypothesis.

We have also noted that sequence variation or conservation can have a profound influence in shaping T cell responses by a different set of mechanisms. In general, we have noted that when individuals are exposed to different strains of the same species, or different species of phylogenetic related microbes, the immune response tends to focus on conserved epitopes. This is because repeated exposure of different but cross-reactive microbes ends up “teasing out” T cell recognizing conserved/homologous epitopes. Specifically, this has been observed in the case of dengue virus (DENV), where repeated exposure to different serotypes focuses the response on conserved epitopes (73, 74). The phenomenon is not limited to viruses, and is also observed in the case of grass pollens and ragweed pollen specific allergic responses (75, 76). In the case of bacterial genomes, we have shown that intragenus conservation within different mycobacteria species shapes T cell responses (10), and epitopes shared between mycobacteria tubercoloid species and other non-pathogenic mycobacteria are preferentially recognized, indicating that differential reactivity may at least partially accounted for by environmental factors. It is currently unknown whether BP antigens and epitopes that share significant homology to other microbes encountered as a result of environmental exposure might be preferentially recognized.

In a separate study, we have recently shown that the sequence similarity between antigens and human microbiome can either dampen or increase T cell epitope immunogenicity (77). In this study, we systematically evaluated the homology of human microbiome sequences and sets of control peptides and T cell epitopes of various autoantigens, allergens, and infectious pathogens. We expected that human adaptive immune system would be largely tolerant toward sequences identical or highly similar to those found in the human microbiome. We therefore predicted that these sequences would be more frequently found in the non-epitope category, as compared to the dominant epitope category. In many instances of epitope categories this was indeed the case, and reactivity was dampened (tolerogenic effect) suggesting that exposure to microbiome-derived sequence homologs might lead to T cell tolerization. However, in other cases, such as for example mycobacteria, and consistent with the studies mentioned above, the reactivity was increased (inflammatory effect) when the epitope sequence was conserved in the microbiome. It is currently unknown whether BP antigens and epitopes that share significant homology to other microbes contained in the human microbiome might be preferentially recognized or conversely tolerized.

## Validation and characterization of T cell epitopes

**Figure 3:**
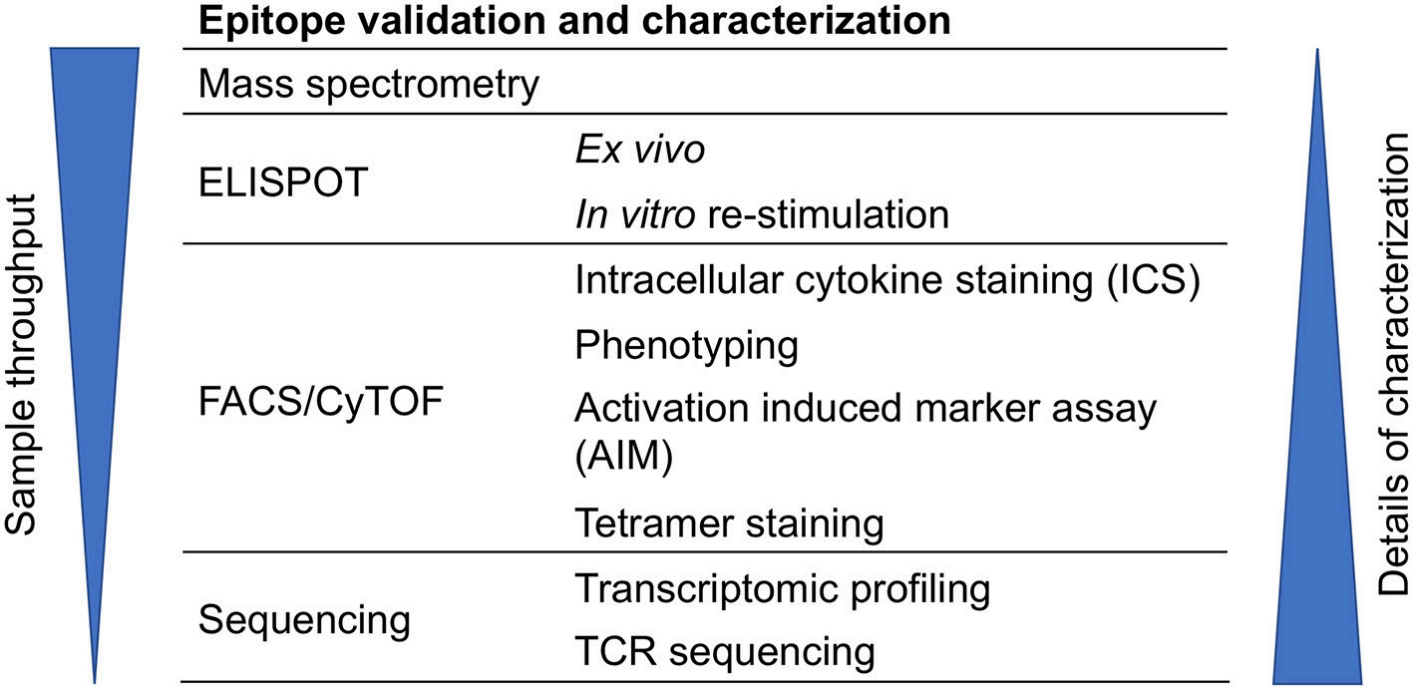
Approaches for epitope validation and characterization. T cell epitopes can be further characterized by various techniques that are based on mass spectrometry, ELISPOT, flow cytometry (FACS), CyTOF, or sequencing.

T cell epitopes can be characterized by various techniques such as mass spectrometry (MS), ELISPOT, intracellular cytokine staining (ICS), activation induced marker (AIM) assay, antigen-reactive T cell enrichment (ARTE) assay, tetramer staining, multidimensional fluorescence-based flow cytometry, and cytometry by time-of-flight (CyTOF), RNA-Sequencing (RNA-seq), and T cell receptor (TCR) sequencing (Figure 3). These techniques can also be combined. For example, performing TCR analysis of tetramer positive cells, or AIM/ARTE assays combined with ICS staining for particular cytokines.

### *In vitro* vs. *ex vivo* characterization

T cell responses can be characterized directly *ex vivo* or after *in vitro* re-stimulation in case that epitope-specific T cells are rare. Though *in vitro* re-stimulation allows for greater sensitivity, it may alter the phenotype of responding T cells; thus, the characterization of re-stimulated T cells require specific adjustments to the experimental strategy. Certain epitope characteristics are not altered by *in vitro* expansion, such as which particular TCR genes are expressed, HLA restriction, sequence conservation of the epitope recognized, or the pattern of cytokine polarization. On the other hand, memory and activation markers and other phenotypic markers usually detected by flow cytometry analysis are altered by the activation caused by cell culture. We have found that it is often possible to assess responses directly *ex vivo*, by using pools of different epitopes or peptides, so that the overall frequency of responding cells is enhanced. This approach is particularly effective when combined with the AIM assay described below, and particularly key to analyze samples with small volume. More specifically, our group has developed a megapool approach, which consists of large numbers of peptides (78). These “megapools” have been utilized in several systems such as allergies (79, 80), tuberculosis (11), tetanus and pertussis (24, 81), and DENV for both CD8 and CD4 T cell epitopes (82–84).

### Mass spectrometry

MS-based approach has been utilized to identify and characterize T cell epitopes presented by MHC molecules since the 1990s (85, 86). Briefly, MHC molecules are purified from cell lysates and their associated peptides isolated and analyzed by MS. Although powerful and widely used, a full discussion of this approach is beyond the scope of the current review. Thus, we refer readers to (87) for more details on MS-based immunopeptidomics.

### ELISPOT and ICS assays

In our experience, ELISPOT assay is the most sensitive and high throughput–friendly method to measure T cell cytokine production. Our group has extensive experience using this method and we routinely utilize ELISPOT as a primary screen. In contrast, ICS assay is better at evaluating T cell phenotype and polyfunctionality. Both ELISPOT and ICS assays can characterize epitope pools even with small amounts of PBMCs. In our hands, we can characterize T cell responses with as little as 1 ml of peripheral blood.

### AIM and ARTE assays

In addition to ICS, the selection of *ex vivo* activated antigen-specific CD4 T cell populations can also be performed by measuring different activation molecules using the AIM assay [e.g., OX40 and CD25, which was co-developed by our group (81, 88)] or ARTE assay. The ARTE approach utilizes magnetic-enrichment of T cells that upregulate CD154 (CD40L) to assess human antigen-specific CD4 T cells *ex vivo* (89). ARTE has been applied to identify antigen-specific T cells for several infections and could select rare antigen-specific T cells after short stimulation period without the need for ICS (90).

### Tetramer staining

This approach identifies antigen-specific T cells using tetramer staining reagents (91, 92). Furthermore, tetramer enrichment technique can be utilized if the frequency of antigen-specific T cells is low (93). However, specific reagents for each unqiue HLA:epitope combination of intertest must be produced in order to use this approach. Thus, it is usually used for in-depth characterization of T cells of selected epitope-specificities and HLA restrictions.

### Multidimensional flow cytometry and CyTOF

These are powerful techniques to characterize cell samples by evaluating the expression of many different markers associated with cell lineages (94), activation and functional activities (95), memory cell subtypes and chemokine receptor expression (96). Multicolor fluorescence-based flow cytometry is in general more user and equipment friendly, as antibodies are more generally available. In addition, this technique allows recovery of the cells by cell sorting, and thus is readily coupled with transcriptomic analysis. In contrast to flow cytometry, CyTOF can detect, discriminate, and quantify antibodies that are conjugated to various heavy-metal isotopes with high accuracy (97). This avoids spectral overlap between fluorophores and allows measuring more cellular parameters simultaneously. High-dimensional phenotypic data can be visualized using algorithms such as visualization of stochastic neighbor embed (viSNE) and spanning-tree progression analysis of density-normalized events (SPADE) (98). We have utilized CyTOF and viSNE to visualize and characterize the heterogeneity of human CD4 effector memory T re-expressing CD45RA (Temra) cells (95).

### Transcriptomic profiling

Epitope-specific T cells can be further characterized in-depth by transcriptomic profiling that uses deep-sequencing technologies, including bulk RNA-seq or single-cell RNA-seq (scRNA-seq). By comparison with bulk RNA-seq, scRNA-seq is a more powerful tool to address cellular heterogeneity and to identify novel subpopulations in a “hypothesis free” manner, since individual cells within the “same” population may differ dramatically (99–101). Gene expression profiling using these methodologies is routinely utilized in our laboratory. Examples include the definition of signatures predictive of latent tuberculosis infection (13), the characterization of CD4 cytotoxic memory T cells (95, 102, 103) or CD4 differential responses to BP primary vaccination after aP boost vaccination (23).

### TCR sequencing

In addition to functional and phenotypic characterization of epitope-specific T cell responses, one can further define their TCR repertoires by TCR sequencing (104). TCRs dictate the antigen specificity of T cells through the interactions with peptide and major histocompatibility complexes. By analyzing epitope-associated TCR repertoires, it is possible to investigate common features of TCRs that are specific for a particular epitope and identify determinants that may predict specificity (105, 106). Thus, this strategy will enable researches to systematically integrate epitopes with their specific TCR sequences as well as their associated T cell responses.

## Genome-wide screen of *Mtb* HLA class II epitopes

As a way to illustrate how the various techniques can be utilized to tackle even large complex microbial genomes, we briefly summarize the results of an *Mtb* genome-wide screen (5). Our general strategy has been to first study in detail a limited number of well characterized dominant antigens, to investigate the mechanisms associated with immunodominance, and provide a point of reference for the genome-wide screen (60). While several dominant antigens were known and well described, a truly systematic and unbiased screen had not been attempted before, due to the complexity of the genome and the large number of ORFs. Next, as summarized below, we performed an unbiased genome-wide screen, and based on the results we selected the dominant epitopes and antigens (9). These were then utilized to characterize the epitope and the phenotype of the associated T cells (8, 10, 11, 11, 14), and also to develop a *Mtb* epitope megapool that was utilized in numerous studies and has proven a valuable tool to analyzed responses in a number of different settings (8, 11, 13, 105).

To perform a genome-wide screen of *Mtb*, we selected all full genome sequences available at that point, and utilized the approaches described above, to define a library of about 20,000 predicted promiscuous binders (5). These were synthetized, and screened first as pools and then in deconvolution experiments to identify the actual epitopes responsible for T cell activation. The library also included over 1,500 different variants not totally conserved amongst the genomes analyzed. Here it could be noted that the capacity to readily test for sequence variants is an advantage of our approach.

We have identified hundreds of different epitopes; the response was thus remarkably broad, and each individual recognized tens of different epitopes, the dominant epitopes, and antigens varied appreciably from one individual to the next. The overwhelming majority of the response was CD4 restricted, which was not unexpected sine the epitopes were identified based on their predicted ability to bind to HLA class II molecules. When the epitopes were mapped back to their antigen of origin using the H37Rv reference genome, a set of 82 antigens were identified as dominant, in that they accounted for about 80% of the total response. The majority of these antigens were not previously identified as T cell antigens.

Further analysis revealed that the vast majority of the response mapped to very discrete regions of the *Mtb* genome, and specifically to three clusters of reactivity within the genome, which encoded close to half of the total reactivity. One of the islands contained the well-characterized antigens early secretory antigenic target 6 kDa (ESAT-6) and culture filtrate protein 10 kDa (CFP10), secreted by Type VII secretion systems (T7SS or Esx system). The other two islands also contained Type VII secretion protein pairs. To further highlight the novelty of these observation, we discovered that the antigens that were recognized as dominant were not limited to the secreted proteins, but also included proteins from the actual secretion apparatus. Thus, the results obtained illustrated the feasibility of the approach, while at the same time identifying a number of novel epitopes and antigens, and providing new insights into the mechanisms of immunodominance.

## The resurgence of BP as a public health concern

BP has been a health concern since the Middle Ages (107), and whooping cough was prevalent and associated with high morbidity and mortality until the widespread vaccination (108). Vaccination with wP vaccine in general population has greatly reduced whooping cough since the 1950s. Nevertheless, the wP vaccine was associated with of minor adverse reactions and very rare serious side-effects, which resulted in its replacement by the aP vaccine in the United States (109, 110). In spite of widespread vaccination, the cases of whooping cough have recently been steadily increasing in the United States (www.cdc.gov). Epidemiological evidence indicates that the increased prevalence may be associated with the switch from wP to aP vaccine in the mid-1990s, further implicating a potential role for waning immunity (www.cdc.gov).

**Figure 4:**
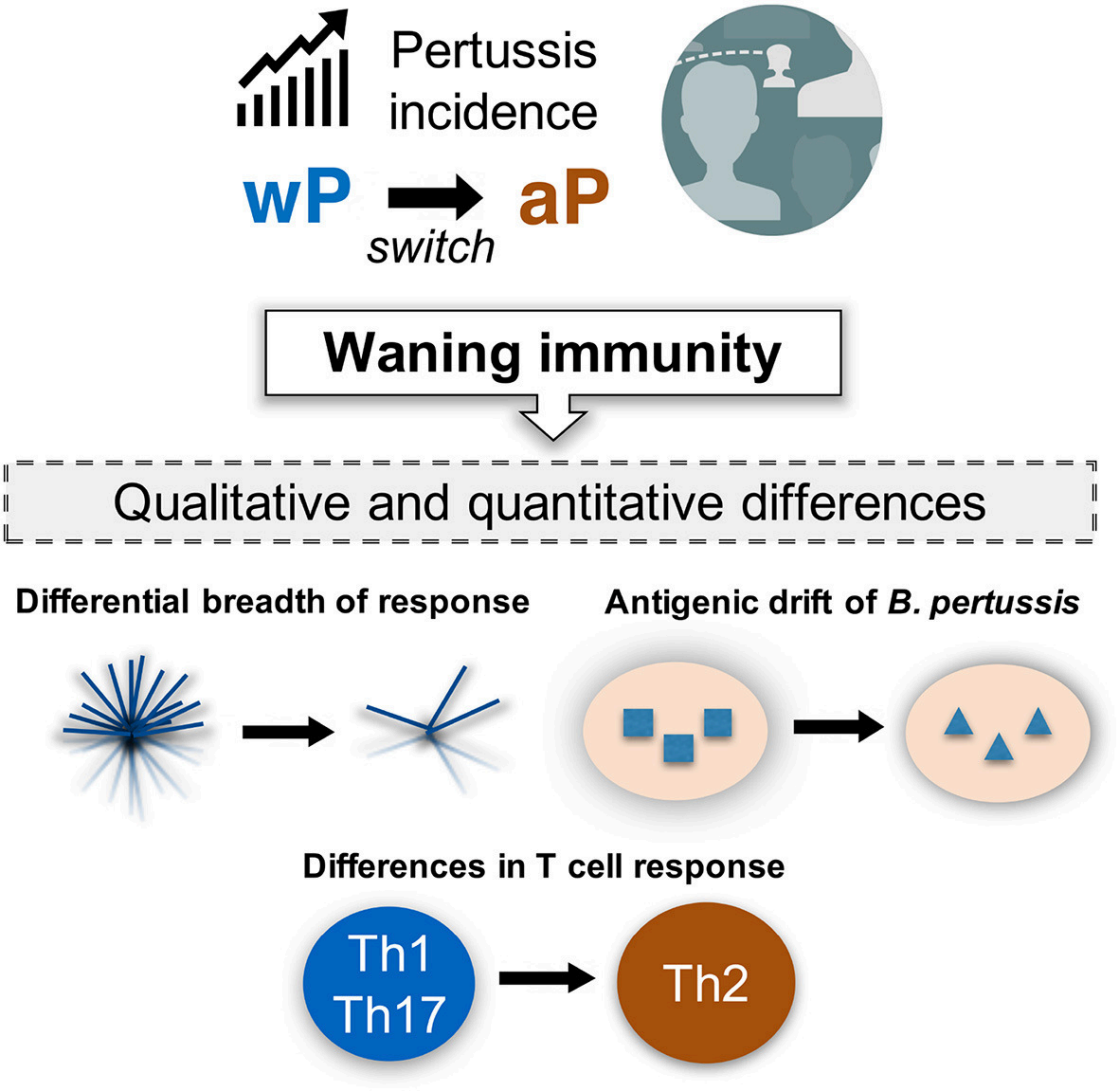
Incidence of pertussis and proposed models of waning immunity. The phenomenon of resurgence of pertussis is gradually increasing as a public health concern, even in countries with high vaccination coverage. It would be important to define the mechanisms associated with waning immunity based on our current knowledge of the qualitative and quantitative changes in both T cell response and BP genetic evolution under vaccine pressure.

Although the phenomenon of “waning BP immunity” is a serious issue (111), it is not straightforward to address as its manifestation appears more than 15 years following the initial vaccination. Therefore, it is crucial to understand the underlying mechanisms of waning immunity in order to guide the design of effective vaccines. In addition to qualitative differences in the response, several other mechanisms may exist. Two main additional hypotheses have been put forth (Figure 4). First, as the wP vaccine contains >3,400 ORFs, whereas the aP vaccine includes only a few BP proteins, it is likely that a differential breadth of response is induced by the wP and aP vaccines (112). Furthermore, the chemically detoxified pertussis toxin (PT) contained in the aP vaccine may have altered antigenicity and could potentially influence vaccine efficacy (113, 114). Second, it has been proposed that decreased vaccine efficacy might be due to antigenic drift (108, 115–119).

Both antibody and T cell responses are thought to be associated with the effectiveness of pertussis vaccination. Notably, protective immunity against BP persists even after antibody levels have reduced (120–122), suggesting that T cells play a role in long-term protection against BP. Animal studies suggest that memory CD4 T cells of Th1 and Th17 phenotype mediate for long-term protection, which are induced by infection as well as wP vaccination (123–125). In contrast, aP vaccination is associated with a predominant Th2 response in humans (126–129). Furthermore, a few studies have reported that aP vaccination induces qualitative changes in T cell responses, resulting suboptimal efficacy (130–133) (Figure 4).

## Genome-wide screen of T cell response to BP

To date, the question of whether the wP vaccine elicits strong T cell responses to additional and different set of antigens to those elicited by aP vaccination, and if so which antigens, has not been addressed. Given the fact that our genome-wide screen of *Mtb* (5) revealed novel dominant antigens that had escaped detection, despite decades of investigation of *Mtb*-specific T cell responses, we consider this possibility likely. By the same token, the breadth of responses induced by natural infection and clinical disease are not known. Here as well, it is likely that additional antigens beyond those included in the current vaccine are of importance; for example, the ACT toxin has been shown to be targeted by BP infected individuals, and the combination of PT and ACT results in superior protection from disease in animal models of BP infection and disease (134–136). These considerations underscore further investigation of BP antigens and T cell epitopes, as well as correlates of protection.

## Definition of aP epitopes following aP vs. wP vaccination

We previously completed a series of studies aimed at the definition of CD4 T cell epitopes derived from the antigens contained in the aP vaccine (24). These illustrate the general feasibility of the study of epitopes and T cell reactivity in BP, and also provide a point of reference to interpret the results obtained in a potential genomic screen of BP T cell reactivity.

In those studies (24), PBMCs from either aP- or wP-primed healthy adults with recent aP booster were used to screen overlapping peptides derived from the protein components that are the foundation of the acellular vaccine: PT, Pertactin, Filamentous hemagluttinin, and Fimbrae 2 & 3). We utilized high-throughput *ex vivo* ELISPOT assays to measure T cell cytokine production of interferon-γ (IFN-γ) and IL-5, and deconvolution of positive peptide pools identified individual T cell epitopes. Epitope mapping revealed the same epitopes were recognized by both aP- and wP-primed individuals (24). However, the ratios of IFN-γ and IL-5 revealed a Th1 bias in originally wP-primed donors, and dominance of IL-5 in individuals primed with aP (24). This differential polarization persists following booster, even decades after original priming (24).

## Characterization and validation of epitopes derived from aP-antigens

As a result of the studies described in the previous section, we defined a “megapool” encompassing the 132 most dominant epitopes recognized, which allowed to assess BP responses directly *ex vivo* using the AIM assay combined with ICS assays, without need for *in vitro* re-stimulation, and thus allow direct phenotyping avoid the alterations induced by the *in vitro* re-stimulation step. This strategy was utilized to evaluate the phenotype and function of T cells in the PBMCs from either wP- or aP-primed donors, following an aP booster 1-3 months post-vaccination, to allow for memory T cells return to steady state conditions (23).

Using the *ex vivo* readouts we still detected the persistent differential polarization previously detected after *in vitro* re-stimulation. Moreover, we detected differential polarization toward IL-4 and IL-9 in aP-primed donors and IFN-γ and IL-17 in wP-primed donors (23). This effect was specific for the vaccine antigens, since no difference was noted for other epitopes such as megapools from the ubiquitous antigens CMV and EBV. The IL-17 polarization of wP vaccination had been previously in baboon models, but not for humans (124, 125, 137, 138). The observation of IL-9 differential polarization is a novel aspect of our study. In-depth phenotypic analysis using combined ICS and transcriptomic analysis of BP-specific memory T cells from aP vs. wP donors revealed clear differences, especially at the level of effector memory T (Tem) cell. 13 differentially expressed genes were identified by comparing ap-Tem and wP-Tem cells, including *IL9* and *TGIF2*, which is related to regulation of TGF-β-responsive genes (139, 140). *IL5, IL13*, and *TGFB1* were also up-regulated in samples from aP donors (23).

In contrast to aP prime, wP prime is associated with substantially higher magnitude of CD4 T cell responses following aP booster, when *ex vivo* responses were assayed in a time window ranging from a few days to several months. Consistent with these findings, by the use of *in vitro* proliferation assays we could show that the aP originally primed donors were associated with lower proliferative capacity (23). In conclusion, these results demonstrate that the various techniques described above can be used to dissect and define the phenotype associated with BP specific T cell responses, and reveal important biological differences.

## Conservation of BP epitopes across BP variants

As mentioned before, previous studies (108, 115–119) indicated that mutation might be accumulating in the acellular vaccine antigens, and that this phenomenon might be related to the apparent waning of BP immunity. Conversely, we also have recently shown that the human microbiome composition modulates T cell responses via molecular mimicry (141).

A first line of preliminary analysis, considers the possibility that new BP strains that carry mutations at key epitopes (pathogen escape), have evolved. Several studies have identified mutations in circulating BP strains that could be the result of pathogen adaptation to immune pressure. For example, Bart et al. (115) identified a total of 471 coding SNPs (genetic variations that result in amino acid changes in the encoded proteins) from pre-vaccination strains. Precise mapping of the T cell epitopes that are prevalently recognized in the human population, including for antigens that are not also targets of immune responses, will further elucidate if the observed genetic variability of circulating BP strains is indeed a result of T cell immune pressure.

## Conclusion and perspective

There is strong evidence suggesting that T cells have important functions in BP immunity and vaccine efficacy. However, T cell epitopes elicited by either natural infection or whole cell (wP) vaccination have not been comprehensively defined, and the corresponding T cell phenotypes have not been characterized. Based upon the success of genome-wide screen of *Mtb*, we make an argument to support performing a genome-wide screen of T cell responses in individuals vaccinated with wP vaccines, and individuals previously diagnosed with whooping cough disease, to understand the targets of cellular immunity in those conditions. Such an investigation could utilize techniques developed and validated over the years, which include both direct *ex vivo* assays such as the AIM assay and *in vitro* expansion of memory T cells utilizing BP lysates. Although powerful, the full genome-wide screen approach also has its limitations. For instance, this approach will not select non-canonical peptides presented by HLA molecules such as peptides originating from non-coding regions and spliced peptides. In fact, recent studies (one of which we coauthored) indicate that a substantial fraction of the HLA peptidome (class I but probably class II as well) is composed of hybrid peptides that originate from two different peptide fragments (so-called cis-spliced or trans-spliced peptides) (142, 143). If general rules that predict the splicing mechanisms can be defined, these sliced peptides could be predicted and thus incorporated in the analysis.

T cell responses against the various epitopes and associated antigens can be characterized and validated using several different complementary approaches. These include determining HLA restriction, and measuring HLA binding affinity, characterizing memory phenotypes, functionality and helper T cell subsets, and patterns of epitope sequence variation. Additionally, it would be of considerable interest to characterize transcriptomic profiles associated with recognition of the new epitopes identified, as compared to the ones currently included in the aP vaccine. These studies could potentially address several hypotheses proposed to explain the decreased efficacy of aP vaccines, namely differences in antigen specificity, differences in functionality, and/or mutations associated with the antigen/epitope associated with vaccine responses. Furthermore, we anticipate that these studies will be broadly applicable to other intracellular bacterial pathogens such as *Salmonella* and *Brucella*.

## Author contributions

All authors listed have made a substantial, direct and intellectual contribution to the work, and approved it for publication.

### Conflict of interest statement

The authors declare that the research was conducted in the absence of any commercial or financial relationships that could be construed as a potential conflict of interest.

## References

[1] Geluk A van Meijgaarden KE de Vries RR Sette A Ottenhoff TH. A DR17-restricted T cell epitope from a secreted *Mycobacterium tuberculosis* antigen only binds to DR17 molecules at neutral pH. Eur J Immunol. (1997) 27:842–7. 10.1002/eji.1830270406 9130633

[2] Stenger S Mazzaccaro RJ Uyemura K Cho S Barnes PF Rosat JP. Differential effects of cytolytic T cell subsets on intracellular infection. Science (1997) 276:1684–7. 10.1126/science.276.5319.1684 9180075

[3] Cho S Mehra V Thoma-Uszynski S Stenger S Serbina N Mazzaccaro RJ. Antimicrobial activity of MHC class I-restricted CD8+ T cells in human tuberculosis. Proc Natl Acad Sci USA (2000) 97:12210–5. 10.1073/pnas.210391497 11035787 PMC17320

[4] Lewinsohn DA Winata E Swarbrick GM Tanner KE Cook MS Null MD. Immunodominant tuberculosis CD8 antigens preferentially restricted by HLA-B. PLoS Pathog. (2007) 3:1240–9. 10.1371/journal.ppat.0030127 17892322 PMC2323292

[5] Lindestam Arlehamn CS Gerasimova A Mele F Henderson R Swann J Greenbaum JA. Memory T cells in latent *Mycobacterium tuberculosis* infection are directed against three antigenic islands and largely contained in a CXCR3+CCR6+ Th1 subset. PLoS Pathog. (2013) 9:e1003130. 10.1371/journal.ppat.1003130 23358848 PMC3554618

[6] Kennedy SC Johnson AJ Bharrhan S Lindestam Arlehamn CS Xu J Garforth SJ. Identification of mycobacterial ribosomal proteins as targets for CD4(+) T cells that enhance protective immunity in tuberculosis. Infect Immun. (2018). 86:e00009–18. 10.1128/IAI.00009-18 29891545 PMC6105890

[7] Johnson AJ Kennedy SC Lindestam Arlehamn CS Goldberg MF Saini NK Xu J. Identification of mycobacterial RplJ/L10 and RpsA/S1 proteins as novel targets for CD4(+) T Cells. Infect Immun. (2017) 85:e01023–16. 10.1128/IAI.01023-16 28115505 PMC5364311

[8] Scriba TJ Carpenter C Pro SC Sidney J Musvosvi M Rozot V. Differential recognition of *Mycobacterium tuberculosis*-specific epitopes as a function of tuberculosis disease history. Am J Respir Crit Care Med. (2017) 196:772–81. 10.1164/rccm.201706-1208OC 28759253 PMC5620682

[9] Carpenter C Sidney J Kolla R Nayak K Tomiyama H Tomiyama C. A side-by-side comparison of T cell reactivity to fifty-nine *Mycobacterium tuberculosis* antigens in diverse populations from five continents. Tuberculosis (2015) 95:713–21. 10.1016/j.tube.2015.07.001 26277695 PMC4666753

[10] Lindestam Arlehamn CS Paul S Mele F Huang C Greenbaum JA Vita R. Immunological consequences of intragenus conservation of *Mycobacterium tuberculosis* T-cell epitopes. Proc Natl Acad Sci USA. (2015) 112:E147–55. 10.1073/pnas.1416537112 25548174 PMC4299226

[11] Lindestam Arlehamn CS McKinney DM Carpenter C Paul S Rozot V Makgotlho E. A quantitative analysis of complexity of human pathogen-specific CD4 T cell responses in healthy M. tuberculosis Infected South Africans. PLoS Pathog. (2016) 12:e1005760. 10.1371/journal.ppat.1005760 27409590 PMC4943605

[12] Okada S Markle JG Deenick EK Mele F Averbuch D Lagos M. IMMUNODEFICIENCIES. Impairment of immunity to Candida and Mycobacterium in humans with bi-allelic RORC mutations. Science (2015) 349:606–13. 10.1126/science.aaa4282 26160376 PMC4668938

[13] Burel JG Lindestam Arlehamn CS Khan N Seumois G Greenbaum JA Taplitz R. Transcriptomic analysis of CD4(+) T cells reveals novel immune signatures of latent tuberculosis. J Immunol. (2018) 200:3283–90. 10.4049/jimmunol.1800118 29602771 PMC5991485

[14] Arlehamn CL Seumois G Gerasimova A Huang C Fu Z Yue X. Transcriptional profile of tuberculosis antigen-specific T cells reveals novel multifunctional features. J Immunol. (2014) 193:2931–40. 10.4049/jimmunol.1401151 25092889 PMC4157075

[15] Lindestam Arlehamn CS Sette A. Definition of CD4 immunosignatures associated with MTB. Front Immunol. (2014) 5:124. 10.3389/fimmu.2014.00124 24715893 PMC3970006

[16] Moguche AO Musvosvi M Penn-Nicholson A Plumlee CR Mearns H Geldenhuys H. Antigen availability shapes T cell differentiation and function during tuberculosis. Cell Host Microbe (2017) 21:695–706 e5. 10.1016/j.chom.2017.05.012 28618268 PMC5533182

[17] Caccamo N Guggino G Meraviglia S Gelsomino G Di Carlo P Titone L. Analysis of *Mycobacterium tuberculosis*-specific CD8 T-cells in patients with active tuberculosis and in individuals with latent infection. PloS ONE (2009) 4:e5528. 10.1371/journal.pone.0005528 19436760 PMC2678250

[18] Tang ST van Meijgaarden KE Caccamo N Guggino G Klein MR van Weeren P. Genome-based *in silico* identification of new *Mycobacterium tuberculosis* antigens activating polyfunctional CD8+ T cells in human tuberculosis. J Immunol. (2011) 186:1068–80. 10.4049/jimmunol.1002212 21169544

[19] Lewinsohn DA Swarbrick GM Park B Cansler ME Null MD Toren KG. Comprehensive definition of human immunodominant CD8 antigens in tuberculosis. NPJ Vaccines (2017) 2:11. 10.1038/s41541-017-0008-6 28775896 PMC5538316

[20] Lindestam Arlehamn CS Lewinsohn D Sette A Lewinsohn D. Antigens for CD4 and CD8 T cells in tuberculosis. Cold Spring Harb Perspect Med. (2014) 4:a018465. 10.1101/cshperspect.a018465 24852051 PMC4066646

[21] Maybeno M Redeker A Welten SP Peters B Loughhead SM Schoenberger SP. Polyfunctional CD4+ T cell responses to immunodominant epitopes correlate with disease activity of virulent Salmonella. PloS ONE (2012) 7:e43481. 10.1371/journal.pone.0043481 22912884 PMC3422266

[22] Cannella AP Tsolis RM Liang L Felgner PL Saito M Sette A. Antigen-specific acquired immunity in human brucellosis: implications for diagnosis, prognosis, and vaccine development. Front Cell Infect Microbiol. (2012) 2:1. 10.3389/fcimb.2012.00001 22919593 PMC3417515

[23] da Silva Antunes R Babor M Carpenter C Khalil N Cortese M Mentzer AJ Th1/Th17 polarization persists following whole-cell pertussis vaccination despite repeated acellular boosters. J Clin Invest. (2018) 128:3853–65. 10.1172/JCI121309 29920186 PMC6118631

[24] Bancroft T Dillon MB da Silva Antunes R Paul S Peters B Crotty S Th1 versus Th2 T cell polarization by whole-cell and acellular childhood pertussis vaccines persists upon re-immunization in adolescence and adulthood. Cell Immunol. (2016) 304–305:35–43. 10.1016/j.cellimm.2016.05.002 PMC4899275 27212461

[25] Sette A Vitiello A Reherman B Fowler P Nayersina R Kast WM. The relationship between class I binding affinity and immunogenicity of potential cytotoxic T cell epitopes. J Immunol. (1994) 153:5586–92. 7527444

[26] Sidney J Steen A Moore C Ngo S Chung J Peters B. Divergent motifs but overlapping binding repertoires of six HLA-DQ molecules frequently expressed in the worldwide human population. J Immunol. (2010) 185:4189–98. 10.4049/jimmunol.1001006 20810981 PMC3307390

[27] Sidney J Steen A Moore C Ngo S Chung J Peters B. Five HLA-DP molecules frequently expressed in the worldwide human population share a common HLA supertypic binding specificity. J Immunol. (2010) 184:2492–503. 10.4049/jimmunol.0903655 20139279 PMC2935290

[28] Southwood S Sidney J Kondo A del Guercio MF Appella E Hoffman S. Several common HLA-DR types share largely overlapping peptide binding repertoires. J Immunol. (1998) 160:3363–73. 9531296

[29] Sidney J Southwood S Moore C Oseroff C Pinilla C Grey HM Measurement of MHC/peptide interactions by gel filtration or monoclonal antibody capture. Curr Protoc Immunol. (2013) Chapter 18:Unit 18 3. 10.1002/0471142735.im1803s100 PMC3626435 23392640

[30] Madden DR. The three-dimensional structure of peptide-MHC complexes. Annu Rev Immunol. (1995) 13:587–622. 10.1146/annurev.iy.13.040195.003103 7612235

[31] Rammensee H Bachmann J Emmerich NP Bachor OA Stevanovic S. SYFPEITHI: database for MHC ligands and peptide motifs. Immunogenetics (1999) 50:213–9. 10.1007/s002510050595 10602881

[32] Sinigaglia F Guttinger M Kilgus J Doran DM Matile H Etlinger H. A malaria T-cell epitope recognized in association with most mouse and human MHC class II molecules. Nature (1988) 336:778–80. 10.1038/336778a0 2462673

[33] Alexander J Sidney J Southwood S Ruppert J Oseroff C Maewal A. Development of high potency universal DR-restricted helper epitopes by modification of high affinity DR-blocking peptides. Immunity (1994) 1:751–61. 10.1016/S1074-7613(94)80017-0 7895164

[34] O'Sullivan D Arrhenius T Sidney J Del Guercio MF Albertson M Wall M. On the interaction of promiscuous antigenic peptides with different DR alleles. Identification of common structural motifs. J Immunol. (1991) 147:2663–9. 1717570

[35] Rothbard JB Lechler RI Howland K Bal V Eckels DD Sekaly R. Structural model of HLA-DR1 restricted T cell antigen recognition. Cell (1988) 52:515–23. 10.1016/0092-8674(88)90464-3 2963699

[36] Krieger JI Karr RW Grey HM Yu WY O'Sullivan D Batovsky L. Single amino acid changes in DR and antigen define residues critical for peptide-MHC binding and T cell recognition. J Immunol. (1991) 146:2331–40. 1706393

[37] Panina-Bordignon P Tan A Termijtelen A Demotz S Corradin G Lanzavecchia A. Universally immunogenic T cell epitopes: promiscuous binding to human MHC class II and promiscuous recognition by T cells. Eur J Immunol. (1989) 19:2237–42. 10.1002/eji.1830191209 2481588

[38] Sette A Sidney J. Nine major HLA class I supertypes account for the vast preponderance of HLA-A and -B polymorphism. Immunogenetics (1999) 50:201–12. 10.1007/s002510050594 10602880

[39] Sette A Sidney J. HLA supertypes and supermotifs: a functional perspective on HLA polymorphism. Curr Opin Immunol. (1998) 10:478–82. 10.1016/S0952-7915(98)80124-6 9722926

[40] Sidney J del Guercio MF Southwood S Engelhard VH Appella E Rammensee HG. Several HLA alleles share overlapping peptide specificities. J Immunol. (1995) 154:247–59. 7527812

[41] Sidney J Grey HM Southwood S Celis E Wentworth PA del Guercio MF. Definition of an HLA-A3-like supermotif demonstrates the overlapping peptide-binding repertoires of common HLA molecules. Hum Immunol. (1996) 45:79–93. 10.1016/0198-8859(95)00173-5 8882405

[42] Sidney J Peters B Frahm N Brander C Sette A. HLA class I supertypes: a revised and updated classification. BMC Immunol. (2008) 9:1. 10.1186/1471-2172-9-1 18211710 PMC2245908

[43] Sidney J Southwood S del Guercio MF Grey HM Chesnut RW Kubo RT. Specificity and degeneracy in peptide binding to HLA-B7-like class I molecules. J Immunol. (1996) 157:3480–90. 8871647

[44] Sidney J Southwood S Mann DL Fernandez-Vina MA Newman MJ Sette A. Majority of peptides binding HLA-A^*^0201 with high affinity crossreact with other A2-supertype molecules. Hum Immunol. (2001) 62:1200–16. 10.1016/S0198-8859(01)00319-6 11704282

[45] Sidney J Southwood S Pasquetto V Sette A. Simultaneous prediction of binding capacity for multiple molecules of the HLA B44 supertype. J Immunol. (2003) 171:5964–74. 10.4049/jimmunol.171.11.5964 14634108

[46] Sidney J Southwood S Sette A. Classification of A1- and A24-supertype molecules by analysis of their MHC-peptide binding repertoires. Immunogenetics (2005) 57:393–408. 10.1007/s00251-005-0004-2 16003466

[47] Southwood S Sidney J Kondo A del Guercio MF Appella E Hoffman S. Several common HLA-DR types share largely overlapping peptide binding repertoires. J Immunol. (1998) 160:3363–73. 9531296

[48] O'Sullivan D Arrhenius T Sidney J Del Guercio MF Albertson M Wall M. On the interaction of promiscuous antigenic peptides with different DR alleles. Identification of common structural motifs. J Immunol. (1991) 147:2663–9. 1717570

[49] Castelli FA Buhot C Sanson A Zarour H Pouvelle-Moratille S Nonn C. HLA-DP4, the most frequent HLA II molecule, defines a new supertype of peptide-binding specificity. J Immunol. (2002) 169:6928–34. 10.4049/jimmunol.169.12.6928 12471126

[50] Berretta F Butler RH Diaz G Sanarico N Arroyo J Fraziano M. Detailed analysis of the effects of Glu/Lys beta69 human leukocyte antigen-DP polymorphism on peptide-binding specificity. Tissue Antigens (2003) 62:459–71. 10.1046/j.1399-0039.2003.00131.x 14617029

[51] Greenbaum J Sidney J Chung J Brander C Peters B Sette A. Functional classification of class II human leukocyte antigen (HLA) molecules reveals seven different supertypes and a surprising degree of repertoire sharing across supertypes. Immunogenetics (2011) 63:325–35. 10.1007/s00251-011-0513-0 21305276 PMC3626422

[52] Sidney J Del Guercio MF Southwood S Sette A. The HLA Molecules DQA1^*^0501/B1^*^0201 and DQA1^*^0301/B1^*^0302 share an extensive overlap in peptide binding specificity. J Immunol. (2002) 169:5098–108. 10.4049/jimmunol.169.9.5098 12391226

[53] Tangri S Mothe BR Eisenbraun J Sidney J Southwood S Briggs K. Rationally engineered therapeutic proteins with reduced immunogenicity. J Immunol. (2005) 174:3187–96. 10.4049/jimmunol.174.6.3187 15749848

[54] Oseroff C Sidney J Kotturi MF Kolla R Alam R Broide DH. Molecular determinants of T cell epitope recognition to the common Timothy grass allergen. J Immunol. (2010) 185:943–55. 10.4049/jimmunol.1000405 20554959 PMC3310373

[55] Oseroff C Sidney J Tripple V Grey H Wood R Broide DH. Analysis of T cell responses to the major allergens from German cockroach: epitope specificity and relationship to IgE production. J Immunol. (2012) 189:679–88. 10.4049/jimmunol.1200694 22706084 PMC3392449

[56] Oseroff C Sidney J Vita R Tripple V McKinney DM Southwood S. T cell responses to known allergen proteins are differently polarized and account for a variable fraction of total response to allergen extracts. J Immunol. (2012) 189:1800–11. 10.4049/jimmunol.1200850 22786768 PMC3411923

[57] Doolan DL Southwood S Chesnut R Appella E Gomez E Richards A. HLA-DR-promiscuous T cell epitopes from Plasmodium falciparum pre-erythrocytic-stage antigens restricted by multiple HLA class II alleles. J Immunol. (2000) 165:1123–37. 10.4049/jimmunol.165.2.1123 10878392

[58] Lamonaca V Missale G Urbani S Pilli M Boni C Mori C. Conserved hepatitis C virus sequences are highly immunogenic for CD4(+) T cells: implications for vaccine development. Hepatology (1999) 30:1088–98. 10.1002/hep.510300435 10498664

[59] Wilson CC Palmer B Southwood S Sidney J Higashimoto Y Appella E. Identification and antigenicity of broadly cross-reactive and conserved human immunodeficiency virus type 1-derived helper T-lymphocyte epitopes. J Virol. (2001) 75:4195–207. 10.1128/JVI.75.9.4195-4207.2001 11287569 PMC114165

[60] Arlehamn CS Sidney J Henderson R Greenbaum JA James EA Moutaftsi M. Dissecting mechanisms of immunodominance to the common tuberculosis antigens ESAT-6, CFP10, Rv2031c (hspX), Rv2654c (TB7.7), and Rv1038c (EsxJ). J Immunol. (2012) 188:5020–31. 10.4049/jimmunol.1103556 22504645 PMC3345088

[61] Assarsson E Bui HH Sidney J Zhang Q Glenn J Oseroff C. Immunomic analysis of the repertoire of t cell specificities for influenza A virus in humans. J Virol. (2008) 82:12241–51. 10.1128/JVI.01563-08 18842709 PMC2593359

[62] Paul S Lindestam Arlehamn CS Scriba TJ Dillon MB Oseroff C Hinz D. Development and validation of a broad scheme for prediction of HLA class II restricted T cell epitopes. J Immunol Methods (2015) 422:28–34. 10.1016/j.jim.2015.03.022 25862607 PMC4458426

[63] Dhanda SK Karosiene E Edwards L Grifoni A Paul S Andreatta M. Predicting HLA CD4 Immunogenicity in Human Populations. Front Immunol. (2018) 9:1369. 10.3389/fimmu.2018.01369 29963059 PMC6010533

[64] Nielsen M Lundegaard C Lund O Kesmir C. The role of the proteasome in generating cytotoxic T-cell epitopes: insights obtained from improved predictions of proteasomal cleavage. Immunogenetics (2005) 57:33–41. 10.1007/s00251-005-0781-7 15744535

[65] McKinney DM Southwood S Hinz D Oseroff C Arlehamn CS Schulten V. A strategy to determine HLA class II restriction broadly covering the DR, DP, and DQ allelic variants most commonly expressed in the general population. Immunogenetics (2013) 65:357–70. 10.1007/s00251-013-0684-y 23392739 PMC3633633

[66] Paul S Dillon MBC Arlehamn CSL Huang H Davis MM McKinney DM. A population response analysis approach to assign class II HLA-epitope restrictions. J Immunol. (2015) 194:6164–76. 10.4049/jimmunol.1403074 25948811 PMC4458389

[67] Paul S Arlehamn CSL Schulten V Westernberg L Sidney J Peters B. Experimental validation of the RATE tool for inferring HLA restrictions of T cell epitopes. BMC Immunol. (2017) 18(Suppl. 1):20. 10.1186/s12865-017-0204-1 28681704 PMC5499093

[68] John M Gaudieri S. Influence of HIV and HCV on T cell antigen presentation and challenges in the development of vaccines. Front Microbiol. (2014) 5:514. 10.3389/fmicb.2014.00514 25352836 PMC4195390

[69] Timm J Walker CM. Mutational escape of CD8+ T cell epitopes: implications for prevention and therapy of persistent hepatitis virus infections. Med Microbiol Immunol. (2015) 204:29–38. 10.1007/s00430-014-0372-z 25537849 PMC4305108

[70] Burdin N Handy LK Plotkin SA. What is wrong with pertussis vaccine immunity? the problem of waning effectiveness of pertussis vaccines. Cold Spring Harb Perspect Biol. (2017) 9:a029454. 10.1101/cshperspect.a029454 28289064 PMC5710106

[71] Diavatopoulos DA Edwards KM. What is wrong with pertussis vaccine immunity? why immunological memory to pertussis is failing. Cold Spring Harb Perspect Biol (2017) 9:a029553. 10.1101/cshperspect.a029553 28289059 PMC5710107

[72] Eberhardt CS Siegrist CA. What is wrong with pertussis vaccine immunity? inducing and recalling vaccine-specific immunity. Cold Spring Harb Perspect Biol. (2017) 9:a029629. 10.1101/cshperspect.a029629 28289058 PMC5710108

[73] Weiskopf D Angelo MA de Azeredo EL Sidney J Greenbaum JA Fernando AN. Comprehensive analysis of dengue virus-specific responses supports an HLA-linked protective role for CD8+ T cells. Proc Natl Acad Sci USA (2013) 110:E2046–53. 10.1073/pnas.1305227110 23580623 PMC3670335

[74] Weiskopf D Angelo MA Bangs DJ Sidney J Paul S Peters B. The human CD8+ T cell responses induced by a live attenuated tetravalent dengue vaccine are directed against highly conserved epitopes. J Virol. (2015) 89:120–8. 10.1128/JVI.02129-14 25320311 PMC4301095

[75] Westernberg L Schulten V Greenbaum JA Natali S Tripple V McKinney DM. T-cell epitope conservation across allergen species is a major determinant of immunogenicity. J Allergy Clin Immunol. (2016) 138:571–8 e7. 10.1016/j.jaci.2015.11.034 26883464 PMC4975972

[76] Pham J Oseroff C Hinz D Sidney J Paul S Greenbaum J. Sequence conservation predicts T cell reactivity against ragweed allergens. Clin Exp Allergy (2016) 46:1194–205. 10.1111/cea.12772 27359111 PMC5007187

[77] Carrasco Pro S Lindestam Arlehamn CS Dhanda SK Carpenter C Lindvall M Faruqi AA. Microbiota epitope similarity either dampens or enhances the immunogenicity of disease-associated antigenic epitopes. PloS ONE (2018) 13:e0196551. 10.1371/journal.pone.0196551 29734356 PMC5937769

[78] Carrasco Pro S Sidney J Paul S Lindestam Arlehamn C Weiskopf D Peters B. Automatic generation of validated specific epitope sets. J Immunol. Res. (2015) 2015:763461. 10.1155/2015/763461 26568965 PMC4629045

[79] Hinz D Seumois G Gholami AM Greenbaum JA Lane J White B Lack of allergy to timothy grass pollen is not a passive phenomenon but associated with the allergen–specific modulation of immune reactivity. Clin Exp Allergy (2016) 46:705–19. 10.1111/cea.12692 26662458 PMC4846575

[80] da Silva Antunes R Pham J McMurtrey C Hildebrand WH Phillips E Mallal S Urinary peptides as a novel source of T cell allergen epitopes. Front Immunol. (2018) 9:886 10.3389/fimmu.2018.00886 29755469 PMC5932195

[81] da Silva Antunes R Paul S Sidney J Weiskopf D Dan JM Phillips E Definition of human epitopes recognized in tetanus toxoid and development of an assay strategy to detect *ex vivo* tetanus CD4+ T cell responses. PloS ONE (2017) 12:e0169086 10.1371/journal.pone.0169086 28081174 PMC5230748

[82] Weiskopf D Angelo MA Grifoni A O'Rourke PH Sidney J Paul S. HLA-DRB1 alleles are associated with different magnitudes of dengue virus-specific CD4+ T-Cell responses. J Infect Dis. (2016) 214:1117–24. 10.1093/infdis/jiw309 27443615 PMC5021234

[83] Weiskopf D Cerpas C Angelo MA Bangs DJ Sidney J Paul S Human CD8+ T cell responses against the four dengue virus serotypes are associated with distinct patterns of protein targets. J Infect Dis. (2015) 212:1743–51. 10.1093/infdis/jiv289 25980035 PMC4633759

[84] Grifoni A Angelo MA Lopez B O'Rourke PH Sidney J Cerpas C. Global assessment of dengue virus-specific CD4+ T cell responses in dengue-endemic areas. Front Immunol. (2017) 8:1309. 10.3389/fimmu.2017.01309 29081779 PMC5646259

[85] Hunt DF Michel H Dickinson TA Shabanowitz J Cox AL Sakaguchi K. Peptides presented to the immune system by the murine class II major histocompatibility complex molecule I-Ad. Science (1992) 256:1817–20. 10.1126/science.1319610 1319610

[86] Cox AL Skipper J Chen Y Henderson RA Darrow TL Shabanowitz J. Identification of a peptide recognized by five melanoma-specific human cytotoxic T cell lines. Science (1994) 264:716–9. 10.1126/science.7513441 7513441

[87] Caron E Kowalewski DJ Chiek Koh C Sturm T Schuster H Aebersold R. Analysis of Major Histocompatibility Complex (MHC) immunopeptidomes using mass spectrometry. Mol Cell Proteomics (2015) 14:3105–17. 10.1074/mcp.O115.052431 26628741 PMC4762616

[88] Dan JM Lindestam Arlehamn CS Weiskopf D da Silva Antunes R Havenar-Daughton C Reiss SM A cytokine-independent approach to identify antigen-specific human germinal center t follicular helper cells and rare antigen-specific CD4+ T cells in blood. J Immunol. (2016) 197:983–93. 10.4049/jimmunol.1600318 27342848 PMC4955771

[89] Bacher P Schink C Teutschbein J Kniemeyer O Assenmacher M Brakhage AA. Antigen-reactive T cell enrichment for direct, high-resolution analysis of the human naive and memory Th cell repertoire. J Immunol. (2013) 190:3967–76. 10.4049/jimmunol.1202221 23479226

[90] Bacher P Scheffold A. Flow-cytometric analysis of rare antigen-specific T cells. Cytometry Part A (2013) 83:692–701. 10.1002/cyto.a.22317 23788442

[91] Mallone R Nepom GT. MHC Class II tetramers and the pursuit of antigen-specific T cells: define, deviate, delete. Clin Immunol. (2004) 110:232–42. 10.1016/j.clim.2003.11.004 15047201

[92] Nepom GT. MHC multimers: expanding the clinical toolkit. Clin Immunol. (2003) 106:1–4. 10.1016/S1521-6616(02)00014-1 12584043

[93] Kotturi MF Swann JA Peters B Arlehamn CL Sidney J Kolla RV. Human CD8(+) and CD4(+) T cell memory to lymphocytic choriomeningitis virus infection. J Virol. (2011) 85:11770–80. 10.1128/JVI.05477-11 21900169 PMC3209290

[94] Burel JG Qian Y Lindestam Arlehamn C Weiskopf D Zapardiel-Gonzalo J Taplitz R. An integrated workflow to assess technical and biological variability of cell population frequencies in human peripheral blood by flow cytometry. J Immunol. (2017) 198:1748–58. 10.4049/jimmunol.1601750 28069807 PMC5296239

[95] Tian Y Babor M Lane J Schulten V Patil VS Seumois G. Unique phenotypes and clonal expansions of human CD4 effector memory T cells re-expressing CD45RA. Nat Commun. (2017) 8:1473. 10.1038/s41467-017-01728-5 29133794 PMC5684192

[96] Weiskopf D Bangs DJ Sidney J Kolla RV De Silva AD de Silva AM. Dengue virus infection elicits highly polarized CX3CR1+ cytotoxic CD4+ T cells associated with protective immunity. Proc Natl Acad Sci USA. (2015) 112:E4256–63. 10.1073/pnas.1505956112 26195744 PMC4534238

[97] Spitzer MH Nolan GP. Mass Cytometry: Single Cells, Many Features. Cell (2016) 165:780–91. 10.1016/j.cell.2016.04.019 27153492 PMC4860251

[98] Diggins KE Ferrell PB Jr Irish JM. Methods for discovery and characterization of cell subsets in high dimensional mass cytometry data. Methods (2015) 82:55–63. 10.1016/j.ymeth.2015.05.008 25979346 PMC4468028

[99] Golumbeanu M Cristinelli S Rato S Munoz M Cavassini M Beerenwinkel N. Single-cell RNA-Seq reveals transcriptional heterogeneity in latent and reactivated HIV-infected cells. Cell Rep. (2018) 23:942–50. 10.1016/j.celrep.2018.03.102 29694901

[100] Kashima Y Suzuki A Liu Y Hosokawa M Matsunaga H Shirai M. Combinatory use of distinct single-cell RNA-seq analytical platforms reveals the heterogeneous transcriptome response. Sci Rep. (2018) 8:3482. 10.1038/s41598-018-21161-y 29472726 PMC5823859

[101] Russell AB Trapnell C Bloom JD. Extreme heterogeneity of influenza virus infection in single cells. Elife (2018) 7:e32303. 10.7554/eLife.32303 29451492 PMC5826275

[102] Patil VS Madrigal A Schmiedel BJ Clarke J O'Rourke P de Silva AD. Precursors of human CD4(+) cytotoxic T lymphocytes identified by single-cell transcriptome analysis. Sci Immunol (2018) 3:eaan8664. 10.1126/sciimmunol.aan8664 29352091 PMC5931334

[103] Tian Y Sette A Weiskopf D. Cytotoxic CD4 T cells: differentiation, function, and application to dengue virus infection. Front Immunol. (2016) 7:531. 10.3389/fimmu.2016.00531 28003809 PMC5141332

[104] Rosati E Dowds CM Liaskou E Henriksen EKK Karlsen TH Franke A. Overview of methodologies for T-cell receptor repertoire analysis. BMC Biotechnol. (2017) 17:61. 10.1186/s12896-017-0379-9 28693542 PMC5504616

[105] Glanville J Huang H Nau A Hatton O Wagar LE Rubelt F. Identifying specificity groups in the T cell receptor repertoire. Nature (2017) 547:94–8. 10.1038/nature22976 28636589 PMC5794212

[106] Dash P Fiore-Gartland AJ Hertz T Wang GC Sharma S Souquette A. Quantifiable predictive features define epitope-specific T cell receptor repertoires. Nature (2017) 547:89–93. 10.1038/nature22383 28636592 PMC5616171

[107] Mills KH. Immunity to *Bordetella pertussis*. Microbes and infection / Institut Pasteur (2001) 3:655–77. 10.1016/S1286-4579(01)01421-6 11445452

[108] Mooi FR NA VDM De Melker HE. Pertussis resurgence: waning immunity and pathogen adaptation - two sides of the same coin. Epidemiol Infect. (2014) 142:685–94. 10.1017/S0950268813000071 23406868 PMC9151166

[109] Fulton TR Phadke VK Orenstein WA Hinman AR Johnson WD Omer SB. Protective effect of contemporary pertussis vaccines: a systematic review and meta-analysis. Clin Infect Dis. (2016) 62:1100–10. 10.1093/cid/ciw051 26908803 PMC4826451

[110] Sheridan SL Frith K Snelling TL Grimwood K McIntyre PB Lambert SB. Waning vaccine immunity in teenagers primed with whole cell and acellular pertussis vaccine: recent epidemiology. Expert Rev Vaccines (2014) 13:1081–106. 10.1586/14760584.2014.944167 25093268

[111] Gu XX Plotkin SA Edwards KM Sette A Mills KHG Levy O. Waning immunity and microbial vaccines-workshop of the national institute of allergy and infectious diseases. Clin Vaccine Immunol (2017) 24:e00034–17. 10.1128/CVI.00034-17 28490424 PMC5498725

[112] Vaughan K Seymour E Peters B Sette A. Substantial gaps in knowledge of *Bordetella pertussis* antibody and T cell epitopes relevant for natural immunity and vaccine efficacy. Hum Immunol. (2014) 75:440–51. 10.1016/j.humimm.2014.02.013 24530743 PMC4792526

[113] Barkoff AM Grondahl-Yli-Hannuksela K Vuononvirta J Mertsola J Kallonen T He Q. Differences in avidity of IgG antibodies to pertussis toxin after acellular pertussis booster vaccination and natural infection. Vaccine (2012) 30:6897–902. 10.1016/j.vaccine.2012.09.003 22981763

[114] Murphy BR Walsh EE. Formalin-inactivated respiratory syncytial virus vaccine induces antibodies to the fusion glycoprotein that are deficient in fusion–inhibiting activity. J Clin Microbiol. (1988) 26:1595–7. 2459154 10.1128/jcm.26.8.1595-1597.1988 PMC266671

[115] Bart MJ van Gent M van der Heide HG Boekhorst J Hermans P Parkhill J. Comparative genomics of prevaccination and modern *Bordetella pertussis* strains. BMC Genomics (2010) 11:627. 10.1186/1471-2164-11-627 21070624 PMC3018138

[116] Octavia S Sintchenko V Gilbert GL Lawrence A Keil AD Hogg G. Newly emerging clones of *Bordetella pertussis* carrying prn2 and ptxP3 alleles implicated in Australian pertussis epidemic in 2008-2010. J Infect Dis. (2012) 205:1220–4. 10.1093/infdis/jis178 22416243

[117] Octavia S Maharjan RP Sintchenko V Stevenson G Reeves PR Gilbert GL. Insight into evolution of *Bordetella pertussis* from comparative genomic analysis: evidence of vaccine-driven selection. Mol Biol Evol. (2011) 28:707–15. 10.1093/molbev/msq245 20833694

[118] Kurniawan J Maharjan RP Chan WF Reeves PR Sintchenko V Gilbert GL. *Bordetella pertussis* clones identified by multilocus variable-number tandem-repeat analysis. Emerg Infect Dis. (2010) 16:297–300. 10.3201/eid1602.081707 20113564 PMC2957989

[119] Mooi FR van Oirschot H Heuvelman K van der Heide HG Gaastra W Willems RJ. Polymorphism in the *Bordetella pertussis* virulence factors P.69/pertactin and pertussis toxin in The Netherlands: temporal trends and evidence for vaccine-driven evolution. Infect Immun. (1998) 66:670–5. 9453625 10.1128/iai.66.2.670-675.1998 PMC107955

[120] Le T Cherry JD Chang SJ Knoll MD Lee ML Barenkamp S. Immune responses and antibody decay after immunization of adolescents and adults with an acellular pertussis vaccine: the APERT Study. J Infect Dis. (2004) 190:535–44. 10.1086/422035 15243929

[121] Dalby T Petersen JW Harboe ZB Krogfelt KA. Antibody responses to pertussis toxin display different kinetics after clinical *Bordetella pertussis* infection than after vaccination with an acellular pertussis vaccine. J Med Microbiol. (2010) 59(Pt 9):1029–36. 10.1099/jmm.0.020826-0 20508003

[122] Heininger U Cherry JD Stehr K. Serologic response and antibody-titer decay in adults with pertussis. Clin Infect Dis. (2004) 38:591–4. 10.1086/381439 14765356

[123] Barnard A Mahon BP Watkins J Redhead K Mills KH Th1/Th2 cell dichotomy in acquired immunity to *Bordetella pertussis*: variables in the *in vivo* priming and *in vitro* cytokine detection techniques affect the classification of T-cell subsets as Th1, Th2 or Th0. Immunology (1996) 87:372–80. 10.1046/j.1365-2567.1996.497560.x 8778021 PMC1384104

[124] Warfel JM Merkel TJ. *Bordetella pertussis* infection induces a mucosal IL-17 response and long-lived Th17 and Th1 immune memory cells in nonhuman primates. Mucosal Immunol. (2013) 6:787–96. 10.1038/mi.2012.117 23187316

[125] Warfel JM Zimmerman LI Merkel TJ. Acellular pertussis vaccines protect against disease but fail to prevent infection and transmission in a nonhuman primate model. Proc Natl Acad Sci USA. (2014) 111:787–92. 10.1073/pnas.1314688110 24277828 PMC3896208

[126] Rowe J Macaubas C Monger TM Holt BJ Harvey J Poolman JT. Antigen-specific responses to diphtheria-tetanus-acellular pertussis vaccine in human infants are initially Th2 polarized. Infect Immun. (2000) 68:3873–7. 10.1128/IAI.68.7.3873-3877.2000 10858197 PMC101661

[127] Rowe J Yerkovich ST Richmond P Suriyaarachchi D Fisher E Feddema L. Th2–associated local reactions to the acellular diphtheria-tetanus-pertussis vaccine in 4- to 6-year-old children. Infect Immun. (2005) 73:8130–5. 10.1128/IAI.73.12.8130-8135.2005 16299307 PMC1307058

[128] Ryan EJ Nilsson L Kjellman N Gothefors L Mills KH Booster immunization of children with an acellular pertussis vaccine enhances Th2 cytokine production and serum IgE responses against pertussis toxin but not against common allergens. Clin Exp Immunol. (2000) 121:193–200. 10.1046/j.1365-2249.2000.01306.x 10931131 PMC1905694

[129] Ausiello CM Urbani F la Sala A Lande R Cassone A Vaccine- and antigen-dependent type 1 and type 2 cytokine induction after primary vaccination of infants with whole-cell or acellular pertussis vaccines. Infect Immun. (1997) 65:2168–74. 9169747 10.1128/iai.65.6.2168-2174.1997 PMC175299

[130] Chiappini E Stival A Galli L de Martino M. Pertussis re-emergence in the post-vaccination era. BMC Infect Dis. (2013) 13:151. 10.1186/1471-2334-13-151 23530907 PMC3623740

[131] Edwards KM Berbers GA. Immune responses to pertussis vaccines and disease. J Infect Dis. (2014) 209 (Suppl. 1):S10–5. 10.1093/infdis/jit560 24158958

[132] Plotkin SA. The pertussis problem. Clin Infect Dis. (2014) 58:830–3. 10.1093/cid/cit934 24363332

[133] van der Lee S Hendrikx LH Sanders EAM Berbers GAM Buisman AM. Whole-cell or acellular pertussis primary immunizations in infancy determines adolescent cellular immune profiles. Front Immunol. (2018) 9:51. 10.3389/fimmu.2018.00051 29416544 PMC5787539

[134] Carbonetti NH Artamonova GV Andreasen C Bushar N. Pertussis toxin and adenylate cyclase toxin provide a one-two punch for establishment of *Bordetella pertussis* infection of the respiratory tract. Infect Immun. (2005) 73:2698–703. 10.1128/IAI.73.5.2698-2703.2005 15845471 PMC1087369

[135] Carbonetti NH. Pertussis toxin and adenylate cyclase toxin: key virulence factors of *Bordetella pertussis* and cell biology tools. Future Microbiol. (2010) 5:455–69. 10.2217/fmb.09.133 20210554 PMC2851156

[136] Boehm DT Hall JM Wong TY DiVenere A Sen-Kilic E Bevere JR. Evaluation of adenylate cyclase toxoid antigen in acellular pertussis vaccines using a *Bordetella pertussis* challenge model in mice. Infect Immun. (2018) 86:e00857–17. 10.1128/IAI.00857-17 30012638 PMC6204743

[137] Warfel JM Edwards KM. Pertussis vaccines and the challenge of inducing durable immunity. Curr Opin Immunol. (2015) 35:48–54. 10.1016/j.coi.2015.05.008 26091979

[138] Warfel JM Zimmerman LI Merkel TJ. Comparison of three whole-cell pertussis vaccines in the baboon model of pertussis. Clin Vaccine Immunol. (2015) 23:47–54. 10.1128/CVI.00449-15 26561389 PMC4711092

[139] Hamid R Brandt SJ. Transforming growth-interacting factor (TGIF) regulates proliferation and differentiation of human myeloid leukemia cells. Mol Oncol. (2009) 3:451–63. 10.1016/j.molonc.2009.07.004 19699159 PMC5527533

[140] Melhuish TA Gallo CM Wotton D. TGIF2 interacts with histone deacetylase 1 and represses transcription. J Biol Chem. (2001) 276:32109–14. 10.1074/jbc.M103377200 11427533

[141] Bresciani A Paul S Schommer N Dillon MB Bancroft T Greenbaum J. T-cell recognition is shaped by epitope sequence conservation in the host proteome and microbiome. Immunology (2016) 148:34–9. 10.1111/imm.12585 26789414 PMC4819143

[142] Liepe J Marino F Sidney J Jeko A Bunting DE Sette A. A large fraction of HLA class I ligands are proteasome-generated spliced peptides. Science (2016) 354:354–8. 10.1126/science.aaf4384 27846572

[143] Faridi P Li C Ramarathinam SH Vivian JP Illing PT Mifsud NA. A subset of HLA-I peptides are not genomically templated: evidence for cis- and trans-spliced peptide ligands. Sci Immunol. (2018) 3:eaar3947. 10.1126/sciimmunol.aar3947 30315122

